# Genotoxic effects of boric acid and borax in zebrafish, Danio rerio using alkaline comet assay

**DOI:** 10.17179/excli2015-404

**Published:** 2015-07-30

**Authors:** Nagihan Gülsoy, Cüneyd Yavas, Özal Mutlu

**Affiliations:** 1 Marmara University, Faculty of Arts and Sciences, Department of Biology, 34722, Goztepe, Istanbul, Turkey; 2 Marmara University, Institute of Pure and Applied Sciences, 34722, Goztepe, Istanbul, Turkey

**Keywords:** boric acid, borax, genotoxicity, Comet assay, Danio rerio

## Abstract

The present study is conducted to determine the potential mechanisms of Boron compounds, boric acid (BA) and borax (BX), on genotoxicity of zebrafish *Danio rerio* for 24, 48, 72 and 96-hours acute exposure (level:1, 4, 16, 64 mg/l BA and BX) in semi-static bioassay experiment. For that purpose, peripheral erythrocytes were drawn from caudal vein and Comet assay was applied to assess genotoxicity. Acute (96 hours) exposure and high concentrations of boric acid and borax increases % tail DNA and Olive tail moment. Genotoxicity was found for BA as concentration-dependent and BX as concentration and time dependent manner. In general, significant effects (P < 0,05) on both concentrations and exposure times were observed in experimental groups. DNA damage was highest at 96 h and 24 h for all BX and BA concentrations, respectively in peripheral blood of *D. rerio*. For the first time, our study demonstrates the effect of waterborne BA and BX exposure on genotoxicity at the molecular level, which may contribute to understanding the mechanism of boric acid and borax-induced genotoxicity in fish.

## Introduction

Boron is naturally present everywhere on the earth, including in rocks, soils, and natural waters. Especially, boron-containing compounds have a significant use in many industries. The largest group is the manufacture of glass, including energy saving insulation fiberglass and borosilicate glasses. Other uses include the manufacture of ceramics, building products, house wares, consumer electronics, fertilizers, alloys, magnets, lubricants, and adhesives (Schubert, 2011[41]). To establish safe environmental regulations for boron compounds are a challenging endeavor due to natural variability in receiving water chemistry that can affect organisms, therefore bioavailability and toxicity.

Boron is an essential micronutrient in plants and nutritionally important in animals and humans. It is also required for the function of various biological processes such as cell structure and enzyme activities, but become toxic at excessive boron levels in the aquatic environment (Ball et al., 2012[8]). Boron enters aquatic environments from both natural and anthropogenic sources. The environmental risks of borates were evaluated by considering exposures resulting from wastewater entering rivers or being used for irrigation and from sewage sludge being applied to agricultural soil. Also, boron compounds could be hazardous to aquatic organisms at concentrations close to the natural environmental level (Schoderboeck et al., 2011[40]). Borates entering the aquatic environment will form undissociated BA and the borate anion. Their solubility defines that borates will be diluted and dispersed throughout the aquatic environment ultimately reaching the sea. The studies of adsorption of BA on soils (Keren and Bigham, 1985[26]) and sediments (Hanstveit et al., 2001[22]) demonstrate that BA is not strongly nor extensively adsorbed to soil or sediment.

Animal species vary in the concentrations associated with deficiency and toxicity. Fish are very sensitive to changes in their environment and play significant roles in assessing potential risk associated with contamination in aquatic environment of compounds. In aquatic species such as rainbow trout (*Oncorhynchus mykiss*), channel catfish (*Ictalurus punctatus*), goldfish (*Carassius auratus*), largemouth bass (*Micropterus salmoides*) and zebrafish (*Danio rerio*) has demonstrated boron deficiencies: embryo-larval development was adversely affected in waters with very low boron concentrations. Birge and Black (1977[9]) reported that LC1 and LC50 for *O.mykiss*, *I. punctatus*, *C. auratus*. Rowe et al. (1998[37]) concluded that embryonic growth of rainbow trout was reduced below 0.1 mg-B/L and that zygote development was affected in zebrafish at concentrations below 0.002 mg-B/L. Zebrafish development was normal at 0.5 mg-B/L. When zebrafish embryos from parent fish exposed to low boron concentrations were placed in 0.5 mg-B/L, development was normal, as embryos appeared able to replenish boron levels. These data demonstrate that extremely low boron concentrations can cause adverse effects through boron deficiency. 

The acute effects on fish are in the range of 10-20 mg-B/L although the quality of these studies was very limited. Other results showed substantially higher values with fish acute values often exceeding 100 mg-B/L. Juveniles and fry appear to be the most sensitive fish life-stage and boron effect on survival, growth and feed intake of fry (Hamilton and Buhl, 1990[21]; Adhikari and Mohanty, 2012[2]). The acute toxicities of boron compounds were examined for Japanese flounder (*Paralichthys olivaceus*) and red sea bream (*Pagrus major*) in terms of fish size and water temperature. In both fish species, the median lethal concentration (LC50) for 96 h of boron increased linearly with increasing fish size and water temperature (Furuta et al., 2007[17]). In zebrafish and mosquitofish, the 96 h median lethal concentrations reported as 14.2 mgB/L and 978 mgB/L respectively (Guhl, 1992[20]; Wallen et al, 1957[56]). The concentration-response curve for boron is likely to be U-shaped for many species, with adverse effects observed at very high and very low concentrations, while no adverse effects are observed at the intermediate concentrations (Lowengart, 2001[29]). The most sensitive tests report that acute effects on fish are in the range of 10-20 mg-B/L. Toxicities of boron compounds on fish and other aquatic organisms including, insect, mollusk etc. have also been reported. Although these studies have provided toxicological assessments of boron, there is still a lack of knowledge about toxicities of boron: data on genotoxicity is still not available.

The DNA damage measured by DNA strand breakage performs as a reliable indicator of genotoxicity. Several studies have shown that the comet assay is so sensitive, rapid and extensively used methods in detection genotoxicity of chemicals under field and laboratory conditions (Abd-Allah et al., 1999[1]; Cavaş and Konen, 2007[12]; Kumar et al., 2010[28]; Cavaş, 2011[11]; Nwani et al., 2011[33]; Pandey et al., 2011[36]; Nagarani et al., 2012[32]; Selvi et al., 2013[42]) in fish.

While the data for humans are plentiful (Oliveira et al., 2001[35]; Çöl and Çöl, 2003[13]; Duydu et al., 2012[14]), boron essentiality has not been conclusively shown in fish, where modes of action and genotoxic affect have not been fully defined. This prompted us to investigate whether boron compounds could affect as a genotoxic agent in fish by testing its influence at different levels BA and BX using isolated fish erythrocytes and on a comet assay system.

## Materials and Methods

Total boron contents were determined by inductively coupled plasma optical emission spectrometer (ICP-OES) in all experimental groups after start of experiment (at the 1 hour). Zebrafish (*Danio rerio*) were purchased from a commercial supplier (Dogasan Aquarium, Istanbul, Turkey) and were transferred to the laboratory. They were acclimated in well aerated tanks for 1 month at 26 ± 1 °C under a 14L:10D photoperiod and were fed with commercial basal diet of *D. rerio* (38,7 % crude protein; 13 % crude lipid; 14,8 % crude fiber ash). The standard water quality parameters were measured at both the beginning and the end of each exposure period using standard methods (APHA, AWWA, WPCF 2005[7]). During the experiment the pH was 7.6-8.1 ± 0,1, the dissolved oxygen level was 8,3 ± 0,3 mg/L, the nitrite was < 0,3 mg /L and ammonium was < 0,05 mg/L). 180 zebrafish of both sexes (mean weight, 0,72 ± 0,1 g and length, 4,7 ± 0,1 cm) were selected and randomly divided into four treatments, and a negative (only aquarium water) and a positive controls (5 mg/L Ethyl methanesulfonate) with three replicate tanks. Each tank (40 × 30 × 50 cm) contained 10 fish with 25 L dechlorinated tap water. Fish were not fed during the experiments.

Two boron compounds: boric acid (H_3_BO_3_, CAS No. 10043-35-3 Merck Millipore, Germany) and borax or sodium tetraborate decahydrate (Na_2_B_4_O_7·_10H_2_O, CAS No. 1303-96-4, Merck Millipore, Germany) were prepared in diluted water and added to the aquariums. Four different concentrations of BA and BX (nominal concentrations: 1 mg/L, 4 mg/L, 16 mg/L and 64 mg/L; actual concentrations: 0,65 ± 0,046mg/L, 3,56 ± 0,25mg/L; 13,84 ± 0,98; 59,36 ± 4,14mg/L) and four time points (24 h, 48 h, 72 h and 96 h) were selected for the acute experiments. Test concentrations were selected based on the acute values of boron compounds to zebrafish, reported as 96-h median lethal concentrations (Guhl, 1992[20]). 

The test water was renewed every 24 h (80 %) to minimize changes due to metabolism by the fish, volatilization of less stable substances and organism catabolites. At the end of exposure periods, bloods were collected by caudal vena on anesthetized fishes (100 mg/L MS-222, Sigma Aldrich) with heparinized syringes and processed for Alkaline Comet analyses. After 5 minute recovery period in well water, fish were replaced in their own aquarium.

The cells were checked for viability before the start of the experiment using Trypan blue dye. The comet assay, detecting DNA strand breaks (single and double strand breaks, and alkali-labile sites), was performed to evaluate BA and BX-induced DNA damage acutely in zebrafish. Following 24, 48, 72 and 96 hours of exposure, two fish were collected from each aquarium at each sampling time and anaesthetized with buffered MS-222. All the experimental procedures were conducted under a yellow lamp in the dark to prevent additional DNA damage. The alkaline comet assay was performed according to the method of Tice et al. (2000[48]) with some modifications. Briefly, the peripheral blood samples were collected from the caudal vasculature using a 1 mL heparinized syringe and the erythrocytes (10 µl) were resuspended in chilled PBS (pH 7.4) buffer. The suspension (65 µl) was then mixed with 100 µl 0.65 % (w/v) normal melting point agarose and placed on a fully frosted slide precoated with a layer of 0.65 % (w/v) high melting agarose. The microscope slide was then immersed in cold (4 °C) lysed solution (2.5 M NaCl, 10 mM Na_2_EDTA, 10 mM Tris-HCl, 1 % SDS pH 10) containing freshly added 1 % Triton X-100, and 10 % DMSO. After 2 h, the slide was incubated in freshly prepared alkaline buffer (1 mM Na_2_EDTA and 300 mM NaOH, pH 13) for 30 min to allow DNA unwinding. Electrophoresis was performed at 4 °C for 30 min at 15 V and 300 mA in the same buffer. The slides were ultimately neutralized with a 0,4 M Tris buffer (pH 7.59) and stained with 75 µL EtBr (10 µg/mL) for immediately observing by fluorescence microscope. Observations were made at a magnification of 400X using an inversion fluorescence microscope (BX51TF, Olympus, Japan) equipped with a 530 nm excitation filter, a 590 nm emission filter, a digital camera (Kameram A640 FL), and a computer-based image analysis system (Kameram Komet Module, Micro System Ltd. Turkey). One hundred cells from each replicate slide were randomly selected for data analysis of DNA damage percentage in the tail, and olive tail moment. The DNA damage percentage was defined as the percentage of DNA damaged cells with more than 5 % damaged DNA in the tail. The Olive tail moment was defined as the product of the percentage of DNA in the tail distribution and the displacement between the head and the tail (Olive et al. 1990[34]). All the slides were analyzed blindly by a single observer to minimize the scoring variability. In this steps, all chemicals were purchased from Sigma-Aldrich.

Experimental data were presented as means ± the standard deviations (SD) of each independent experiments performed in duplicate. All statistical analyses were performed using SPSS for Windows version 11.0 (SPSS Inc., Chicago, IL, USA). A three-independent-samples test was used to analyze the difference between groups, followed by the non-parametric Mann-Whitney U test. Significance was ascribed at P < 0.05.

## Results and Discussion

Boron enters the aquatic environment from various sources, including weathering of borates, sewage effluents, coal combustion, use of cleaning compounds, and agrochemicals. In the present study was designed to generate data on acute genotoxic study of different BA and BX concentrations on zebrafish for the first report. 

Zebrafish were exposed to nominal BA and BX concentrations ranging from 1 to 64 mg/L (Table 1(Tab. 1)). BA and BX concentrations in the control medium were below the ICP-AES detection limit (0.5 µg/L). It is assumed that 95-100 % of BA and BX present in the experimental media because every day the test media was changed (80 %) with fresh media which is consistent with the increase of BA and BX accumulation in zebrafish after 24, 48, 72 and 96 h of exposure. Cell viability measured at the time of the experiment always exceeded 95 % in all the treatment groups.

**Figure 1:**
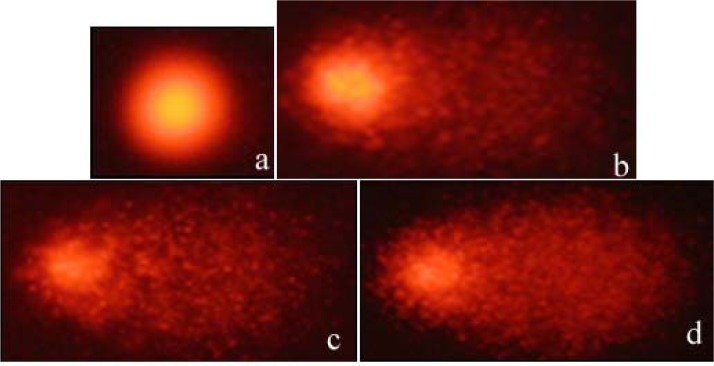
Erythrocytes of *D. rerio* showing: (a) negative control DNA, (b) positive control DNA, (c) DNA damaged after exposure to boric acid (BA) and (d) DNA damaged after exposure to borax (BX)

**Figure 2:**
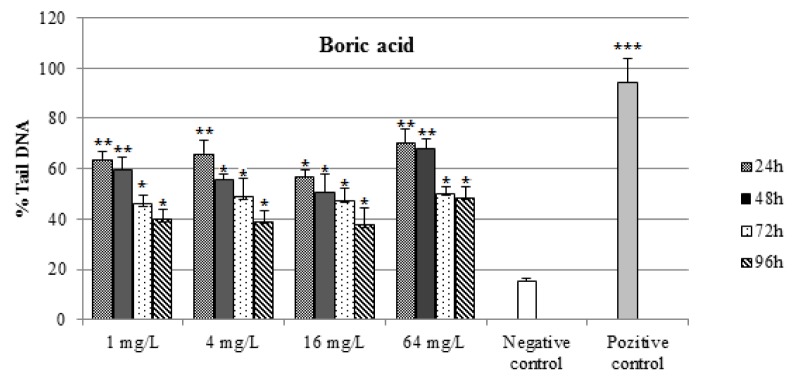
Percentages of comet tail DNA in erythrocytes of *D. rerio* water-borne boric acid concentration and control groups. n=10 for each concentration/duration group ^***^P < 0.001 , ^** ^P < 0,01, ^*^P < 0.05.

**Figure 3:**
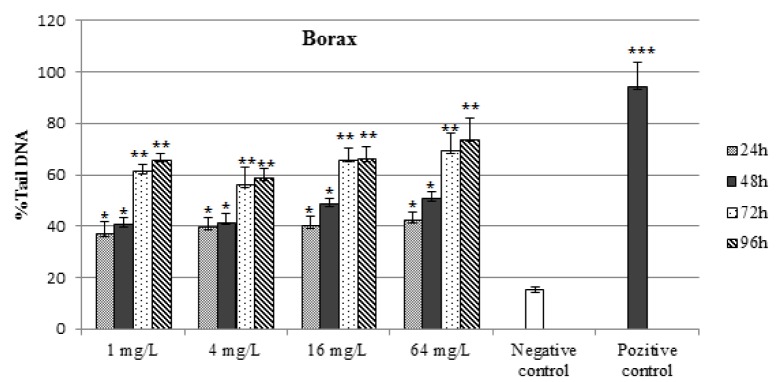
Percentages of comet tail DNA in erythrocytes of *D. rerio* water-borne borax concentration and control groups. n=10 for each concentration/duration group ^***^P < 0.001 , ^** ^P < 0,01, ^*^P < 0.05.

The DNA damages measured as percentage tail DNA and Olive tail moment in the peripheral blood of controls and exposure groups. By performing the comet assay with erythrocytes (Figure 1(Fig. 1)), we found that the specimens that were exposed to all concentrations of BA and BX had an significant increase in DNA damage compared to negative control (15. 36 ± 1.12 % Tail DNA and 0.161 ± 0.05 Olive tail moment, P < 0,01 and P < 0,05 in both comparisons). When compare between the pozitive control (94.03 ± 9.71 % Tail DNA and 35,69 ± 1.8 Olive tail moment) and experimental groups, we found high comet scores (Table1(Tab. 1)). All results of comet assay experiments are summarized in Table 1(Tab. 1) and in Figures 1-3(Fig. 1)(Fig. 2)(Fig. 3).

As shown in Figures 1(Fig. 1) and 2(Fig. 2), DNA damage percentage, % Tail DNA and Olive tail moment in the comet assay increased significantly with increasing BA and BX doses in a dose-dependent manner. In BA group, The DNA strand breaks were not found to be time-dependent and the highest DNA damage was observed 64 mg/L after exposure 24 h as 70.28 ± 5.62 % Tail DNA and 10.97 ± 1.11 Olive tail moment. The other BA exposure groups were shown lower DNA damage than the highest concentration. However when the concentration increase, the DNA damages not linearly increased in all exposure groups. In BX group, the DNA damage was found to be concentration-dependent in erythrocytes, with the highest damage at 64 mg/L concentration, followed 16 mg/L, 4 mg/L and 1 mg/L. The highest DNA damage was observed on 96 h in blood cells (73,81 ± 8.42 % Tail DNA and 11.74 ± 1.68 Olive tail moment) at the highest concentration. The lowest DNA damage was detected at 24 h followed by linear increase. The highest DNA damage was seen on 96 h in all treatment groups. With the respect to effect of duration on DNA, a significant effect (P < 0.01) in blood cells was also observed in fish exposed to various BX concentrations.

To date, many chemicals involved in the environment have identified in fish as genotoxic but there is no result of boron compounds. In the recent years, several studies have been reported by the Comet assay (Abd-Allah et al., 1999[1]; Andrade et al., 2004[6][5]; Buschini et al., 2004[10]; Jha, 2008[24]; Frenzilli et al., 2009[16]) to evaluated the genotoxic agents in various species of fish (Sumathi et al., 2001[45]; Akcha et al., 2003[4]; Cavaş and Konen, 2007[12]; Simoniello et al., 2009[43]; Kumar et al., 2010[28]; Scalon et al., 2010[39]; Yang et al., 2010[57]; Cavaş, 2011[11]; Nwani et al., 2011[33]; Pandey et al., 2011[36]; Nagarani et al., 2012[32]; Selvi et al., 2013[42]) and aquatic invertebrates which can serve as excellent source of material for genotoxic, mutagenic and carcinogenic studies on environment.

In ecotoxicological studies, the fishes are used as sentinel organisms and they have a number of roles in trophic web, accumulate toxic substances and respond to low concentration of mutagens (de Andrade et al., 2004[6][5]; Jha, 2008[24]; Soucek et al., 2011[44]). Zebrafish has been also used as model organism in genotoxic studies especially during embryo development. This study indicates that the acute genotoxicity of BA and BX to *D. rerio *and our results showed that ambient boron compounds could pose a genotoxic agent for fish erythrocytes.

Most of the studies reported that boron compounds were found to be non-genotoxic and even boron had antioxidant effects on various human cell lines *in vitro* and rat tissues (Türkez et al., 2007[53], 2010[54], 2012[52][50], 2013[51]; Türkez, 2008[49]; Geyikoğlu and Türkez, 2008[18]; Ince et al., 2014[23]; Üstündağ et al., 2014[55]). In these studies BA and BX were generally used protector agent for metals or drugs induced genotoxicity *in vitro*. In mammals, it is well known that boron diffuses passively in the body and is not likely to be metabolized according to thermodynamic action. Therefore for induction of DNA damage in mammalian cells the much higher dose levels is reguired (Muller and Kasper, 2000[31]). In these studies, administered boron compounds were applied very short period (1-2 hours) and used low concentration levels (1,25-5mg/L) *in vitro*. The information of such limitations was assumed *in vitro* conditions are not enough for showing genotoxic potential of boron compounds separately.

Boron toxicity is argued in many studies, but none of them related that the environmental sentinel and non-target organisms. Konuk et al. (2007[27]) reported that boron treatment significantly and dose-dependently inhibited the mitotic index and mitotic phase frequencies on *Allium cepa* root meristem cells. On the other hand, Kekec et al. (2010[25]) showed that the genotoxic effects of BA at ranging from 5-150 ppm on wheat by RAPD analysis. Similarly, Sakcali et al. (2013[38]) observed the DNA methylation on maize after boron exposure at the same concentrations. 

The most sensitive taxonomic group to the boron was fish and there was the high variation in LC_50_ within this group (Schoderboeck et al., 2011[40]). In their discussion, the authors concluded that *Brachydanio rerio* had 1.8 mg B/L as the no-observed effect concentration (not published) and boron compounds could be hazardous to aquatic organisms at concentrations close to the natural environmental background in some regions. Taylor et al. (1985[46]) reported the 96 h LC50 values for boron were 74 mg/L for dab, *Limanda limanda*, Thompson et al. (1976[47]) 43 mg/L for 1.8-3.8 g coho salmon, *Oncorhynchus kisutch*. Eckhert (1998[15]) reported boron is not toxic to fish of the genus *Oncorhynchus* at concentrations < 10 mg/L. Wallen et al. (1957[56]) observed that the 96 h LC50 values for boron were as high as 408 mg/L as sodium tetraborate and 979 mg/L as BA for the mosquito fish (*Gambusia affinis*). Furuta et al. (2007[17]) found that that the 96 h LC50 values of boron increased linearly with increasing fish size, ranging from 108 to 252 mg B/L for the flounder, *Paralichthys olivaceus* and from 97 to 172 mg B/L for the sea bream, *Parus major*. Soucek et al. (2011[44]) showed that the fathead minnow (*Pimephales promelas*) is more acutely sensitive to boron than the other aquatic species (an insect, two crustaceans, and four bivalve mollusks). Adhikari and Mohanty (2012[2]) indicates that *C. mrigala* fry exposed BX at 8.0 mg/L reduced growth and feed intake when compared with controls. 

Boron toxicity to aquatic animals is a function of its form and the species of organism and its life stage (Birge and Black, 1977[9]). BA is the predominant and most available form of boron in freshwater ecosystems (pH 6-9) (Goldberg, 1997[19]), and also Birge and Black, (1997[9]) conducted many studies to determine the toxicity of BX to a variety of organisms. These studies indicated that BA and BX toxicity to the same species may differ significantly. In this study the maximum increase in % tail DNA was observed at 24 h and reductions in % tail DNA were seen at 48, 72 and 96 h. The 96 h value had still high from negative control level at all doses. This limited decrease indicated that cytoprotective and tolerance mechanisms or repair of damaged DNA in the cell. In BX group, maximum increase in % tail DNA was seen at 96 h. 24, 48 and 72 h values were also above the negative control and showed gradual increase in all time points. Our genotoxic data indicated that BA and BX has different mode of action and BX is more steadily efficient of *D. rerio* erythrocytes so it should be monitored carefully in the aquatic environment. We conferred this susceptibility might due to zebrafish have more sensitive organism for toxic substance. But since there is no information on genotoxic effects of boron to fish, further studies should be conducted to understand the genotoxic effects and mechanisms of boron compounds to fish.

## Conclusion

DNA strand breakage induced by boric acid and borax, regardless of dose and time depend, could be detected by the Comet assay in fish in the aquatic environment, especially near the boron rich areas. The sensitivities to boron compounds among organisms are common. Therefore genotoxic potential of these compounds should be investigated by the field and the laboratory controlled experiments. Our study suggested that the findings reported herein should help elucidate the respective significance and detect the levels of boric acid and borax in environment because of their genotoxic effects.

## Acknowledgements

This work was supported by the Marmara University (BAP Fen-A-060510-0139).

## Conflict of interest

The authors declare that they have no conflict of interest.

## Figures and Tables

**Table 1:**
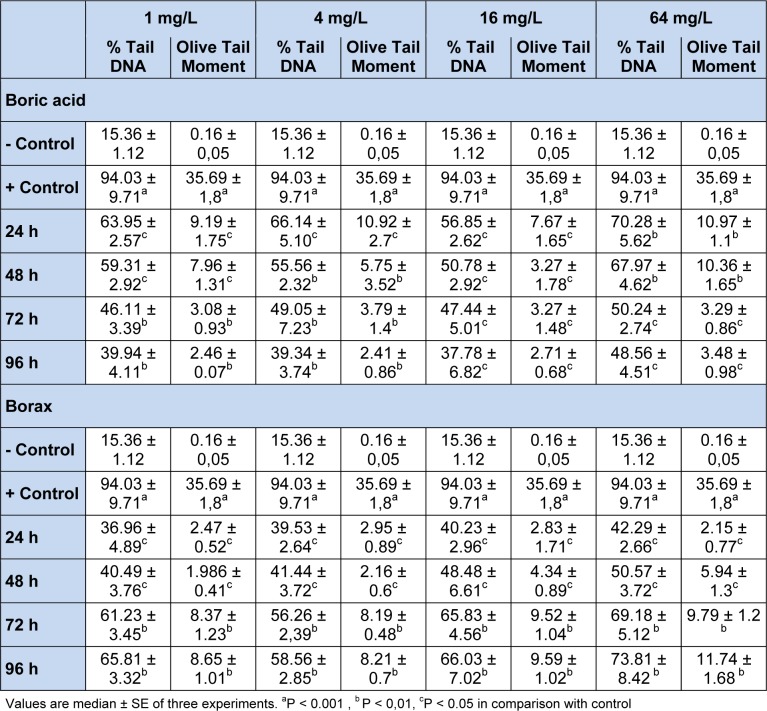
Effect of different concentrations of boric acid and borax on comet parameters in the erythrocytes of *D. rerio*
